# Respirable silica particles in coal mine dust: An image library dataset collected using scanning electron microscopy with energy dispersive X-ray spectroscopy

**DOI:** 10.1016/j.dib.2023.109656

**Published:** 2023-10-09

**Authors:** Çiğdem Keleş, Emily Sarver

**Affiliations:** Department of Mining and Minerals Engineering, Virginia Tech, Blacksburg, VA 24061, USA

**Keywords:** Respirable crystalline silica, Occupational health, Silicosis, Coal workers pneumoconiosis, SEM-EDX

## Abstract

This dataset comprises an image library of 282 respirable silica particles. The particles were identified in samples of respirable coal mine dust (RCMD) collected in numerous US underground mines, and samples of lab-generated respirable dust that were created using the primary dust source materials (e.g., raw coal and rock) obtained from those mines. (A limited number of particles were also identified in samples generated from silica-containing reference materials.) Silica particle identification was done by scanning electron microscopy with energy dispersive X-ray spectroscopy (SEM-EDX), and then each particle was imaged and analyzed at both low (5 kV) and high (20 kV) accelerating voltage. SEM micrographs were captured at high magnification (i.e., 5000-20,000 ×) and overlaid with elemental maps to visually indicate relative Si and Al content; spectra were also collected to determine Si and Al % in each particle. This dataset can inform the understanding of real respirable silica particles in coal mine environments, which may differ from idealized (i.e., pure, independent) silica particles. The dataset therefore provides valuable context for the design and interpretation of research related to: respirable silica exposure studies, sample analysis and monitoring techniques, or dust control.

Specifications Table

**Table:** 

Subject	Occupational health
Specific subject area	Respirable silica particles in underground coal mine environments
Type of data	Table Image
How the data were acquired	All data were acquired by scanning electron microscopy with energy dispersive X-ray spectroscopy (SEM-EDX), using an FEI Quanta 600 FEG environmental SEM (FEI, Hillsboro, OR) equipped with a Bruker Quantax 400 EDX spectroscope (Bruker, Ewing, NJ) and Esprit software (Version 1.9.4). In total, the dataset represents 282 respirable silica particles contained in 61 dust samples. For each particle, the following data were captured at both 5 and 20 kV accelerating voltage: SEM images (i.e., micrographs) with elemental maps and normalized atomic % values for eight elements (Si, Al, C, O, Ca, Mg, Fe, Ti).
Data format	Raw Analyzed
Description of data collection	Respirable silica particles included in this dataset were identified in three types of dust samples: (1) Respirable coal mine dust (RCMD) samples that had been previously collected in a group of underground mines; and respirable samples that had been lab-generated from (2) bulk dust-source materials obtained from the same group of mines and (3) reference materials. These samples were associated with a prior study of respirable silica sources and characteristics in coal mines [1,2], but the current dataset represents separate follow-up work to create an extensive image library.
Data source location	Descriptions were previously published [1,2] of the mines from which the RCMD samples and bulk dust-source materials originated. While exact locations/mine identities are not available, the published descriptions include geographic regions and general information related to geology and mining conditions.
Data accessibility	Repository name: Mendeley Data Data identification number: DOI: 10.17632/w5yjhpkybx.2 Direct URL to data: https://data.mendeley.com/datasets/w5yjhpkybx

## 1 Value of the Data

• The composition of respirable coal mine dust (RCMD) can vary considerably, but silica is often one component. Chronic exposure RCMD can lead to a number of lung diseases, including coal workers pneumoconiosis (CWP or “black lung”); and the silica component, (which is typically present at crystalline silica), specifically, is linked to the most severe form of CWP called progressive massive fibrosis (PMF).
• While silica content in RCMD is typically monitored on the basis of mass percentage, particle characteristics are increasingly recognized as important drivers for lung deposition, translocation, and response. However, there remains a disconnect between conceptual models that assume idealized silica (i.e., pure, independent) particles and real silica particles as they occur in the occupational environment, which may vary in composition (e.g., from pure, to impure, to occluded), morphology (e.g., size and shape) and association (i.e., independent, agglomerated).
• This dataset provides visual evidence of a range of respirable silica particle types observed directly in RCMD samples from numerous mines, as well as in dust samples generated in the laboratory using primary source materials from the same mines (e.g., raw coal and rock) and several reference materials. Elemental data collected at two different accelerating voltages can also support interpretation of particle surfaces.
• The dataset can benefit anyone interested in the characteristics of respirable silica particles, including researchers, clinicians, engineers, and policymakers. More broadly, the data collection techniques might be of interest to those exploring the composition of particle surfaces.
• The dataset can inform future studies aimed at: elucidating health effects associated with occupational exposures to respirable silica; development of respirable silica analytical techniques or real-time monitoring devices; and evaluation of dust control technologies and practices.

## 2 Objective

The dataset was gathered following a prior research study of respirable silica sources and particle characteristics in underground coal mines [1,2]. That study included SEM-EDX analysis of particles in RCMD samples from 15 US mines, as well as in respirable dust samples that were laboratory-generated from primary dust-source materials (e.g., raw coal and rock) obtained from the same mines. During that SEM-EDX work, it was generally observed that respirable silica particles^1^ had a range of occurrence modes—from relatively pure silica to clay-occluded silica or silica ingrained in another type of particle, and from independent silica particles to those in micron-sized agglomerates—however, only a limited number of images were collected [2].The objective of this follow-up effort was thus to compile an image library to document respirable silica particles in the RCMD samples and in the lab-generated samples of source materials. The dataset also includes images of respirable silica in samples generated from several reference materials.

## 3 Data Description

The dataset is available with open access: https://data.mendeley.com/datasets/w5yjhpkybx. It is essentially a library of images and associated elemental data for 282 respirable silica particles, which were identified 61 samples (Table 1). The samples were of three types:

(1) RCMD: respirable coal mine dust samples collected in standardized locations in numerous underground US mines (numbered 10–24). The locations include: intake, production, roof bolter, feeder breaker, and return.
(2) Source material: respirable dust samples generated in the laboratory from primary dust-source materials obtained from the same group of mines. Source material types include run-of-mine (ROM) coal or ROM rock (which are raw coal and rock being produced by the mine), bolter dust (which is material take directly from the dust collection system of a roof bolter), and rock dust (which is the inert stone dust that is applied in underground coal mines to mitigate explosion hazards).
(3) Reference material: respirable dust samples generated in the laboratory from reference materials. Reference material types include Min-U-Sil 5 or Sil-Co-Sil (US Silica, Katy, TX; these are high purity quartz products), VCAS-160 (Vitro Minerals, Jackson, TN, USA; this is a high purity aluminosilicate glass powder), and Ball Clay (The Ceramic Shop, Norristown, PA, USA; this is an aluminosilicate powder that has moderate silica content).

**Table 1:** Number of respirable silica particles per sample type. For RCMD samples, the mine and sampling location are indicated; for samples generated from mine dust-source materials or reference materials, the material type is shown.

Sample Type:	RCMD Samples					Samples from Source Materials				Samples from Reference Materials			
Location/Material:	Intake	Production	Bolter	Feeder	Return	ROM coal	ROM rock	Bolter dust	Rockdust	Min-U-Sil 5	Sil- Co-Sil	VCAS- 160	Ball Clay
Mine 10	5			5	4		4			4	4	4	4
Mine 11		2											
Mine 12		5				6							
Mine 13	2				2			5					
Mine 14				4		3							
Mine 15		6	3		4	4	3						
Mine 16						4		8	2				
Mine 17	4		11					4	2				
Mine 18			4			4	5	9					
Mine 19		4	6	6	5	4	6	6	1				
Mine 20									1				
Mine 21	4		4	4		4	6	9					
Mine 22		4		4		3	6	3	3				
Mine 23						4	14	7	2				
Mine 24	5				7			7	3				
Total	20	21	28	23	22	36	44	58	14	4	4	4	4

	114					152				16			
282												

For each respirable silica particle included in the image library, the following data are tabulated: sample type and identifiers (i.e., mine number and sampling location for RCMD samples, mine number and material type for samples generated from source materials, or material type for reference materials); particle number; magnification for SEM-EDX analysis; SEM images with EDX-derived elemental maps (Si and Al) captured at 5 and 20 kV; and Si and Al atomic % values measured at 5 and 20 kV. Table 2 illustrates the data presentation format for just eight particles, but the full *Respirable silica particle image library* table includes the same format for all 282 particles. Additionally available is a table of *Normalized atomic % values* for all eight elements included in the particle analysis (i.e., Si and Al, plus C, O, Ca, Mg, Fe and Ti); it includes 564 rows of data (i.e., values for 282 particles at both 5 and 20 kV).

**Table 2:** Data presentation format for the respirable silica particle image library. For each particle, a pair of images (captured at 5 and 20 kV) is shown with Al and Si maps overlaid; additionally, the sample identifiers and the particle number are reported, along with the SEM magnification and the measured Al and Si % at 5 and 20 kV. If only one silica particle in the image was analyzed, the image is centered on that particle; if multiple particles were analyzed, the particle for which elemental values are reported is marked with +. (Images with reddish background indicate that Al signal across the entire mapped field was essentially noise, i.e., Al content in the particle(s) was no higher than in the background.)

## 4 Experimental Design, Materials and Methods

### 4.1 Respirable dust samples

As noted above, respirable silica particles were identified in three different types of samples. These samples were part of larger sample sets that had already been collected and analyzed for prior study [1,2]. The sections below outline key details regarding sample collection, and the approach to selecting a subset of the available samples for inclusion in this effort to build a respirable silica image library.

#### 4.1.1 RCMD samples

RCMD samples were collected from 15 underground mines in the US (Mines 10–24). At the time of sample collection, each mine was operated by an industry partner, which provided mine access and logistical support for sampling. A detailed description of the mines and sampling procedures can be found in [1,2]. Briefly, samples were collected in five standardized locations: in the intake (clean) airway to an active mine section, just downwind from the active production face, just downwind from an active roof bolter, adjacent to the raw material feeder breaker, and in the return (exhaust) airway of an active mine section. The sampling train consisted of an air pump, a 10-mm nylon cyclone, and two-piece styrene filter cassette loaded with a polycarbonate (PC) filter (37-mm, track etched with nominal 0.4 µm pore size) and cellulose support pad. The pump was operated at 2.0 L/min such that the cyclone penetration efficiency approximates the ISO “respirable convention” (i.e., maximum aerodynamic diameter of approximately 10 µm, 50 % cut point of approximately 4 µm) [3]. Samples were generally collected over a continuous period of 2–4 h.

While a total of 75 RCMD samples were collected and analyzed as part of a prior study [1,2], only 25 (per Table 1) were selected for the follow-up effort to compile the respirable silica image library reported here. RCMD sample selection for this effort was generally done to enable efficient SEM-EDX work to identify and analyze silica particles (described below). Selection was based on two main criteria: the sample had to have (1) sufficient silica content (i.e., more than about 3%), and (2) a favorable particle loading density on the sample filter (i.e., between about 0.01-0.035 particles/µm^2^). (The silica content and particle loading density data were reported in [2].) Moreover, an effort was made to ensure that selected samples represented all five sampling locations and most of the 15 mines.

#### 4.1.2 Laboratory-generated samples

Mine dust-source materials. From the 15 mines where RCMD samples were collected, bulk samples of four types of dust-source materials were also obtained: run-of-mine (ROM) coal and rock, which are the raw geologic material that are mined at the production face and were hand-picked off of the main production conveyor belt; material take directly from the dust collection system on the roof bolter machine, which essentially vacuums dust generated by the bolter as it drills holes for roof bolt installation; and rock dust products, which are typically high quality limestone or dolostone powder products that are applied to mine surfaces (i.e., floor, walls) to mitigate explosion hazards. As described in [1,2], these four material types are the primary sources of respirable dust in most underground coal mines in the US; and while silica in RCMD is most often associated with the rock strata mined with the target coal (i.e., ROM rock) or drilled for roof bolting (i.e., bolter dust), silica particles can also be source from the coal seam itself (i.e., ROM coal) or the applied rock dust products.

The bulk bolter dust and rock dust product materials were already powdered, however the ROM coal and rock were very coarse as collected from the mine (i.e., >25 mm). Thus, the ROM materials were pulverized and sieved (dry) to <63 µm prior to generation of respirable dust samples.

Respirable samples of each source material were generated in the laboratory in a small enclosure (approximately 0.5 m x 0.5 m x 0.5 m). Briefly, a small mass of the powdered material was placed inside the enclosure and aerosolized using pulses of compressed high-purity air. The respirable-sized particles were collected using the same sampling train as described above for RCMD sampling, such that particles were deposited directly on the PC filter. Given the high concentration of dust inside the enclosure, sampling times were on the order of minutes rather than hours.

A total of 46 bulk source materials were obtained, and respirable samples were generated from each, for the preceding study of silica sources [1]. However, only 32 of those samples (per Table 1) were selected to build the silica image library. Like for the RCMD samples, selection for this follow-up work was based on silica content and particle loading density on the sample filter (as reported by [1]), as well as an effort to ensure representation across material types and mines.

Reference materials. In addition to the bulk dust-source materials from mines, four reference materials were also used to generated respirable dust samples in the laboratory: Min-U-Sil 5 and Sil-Co-Sil (US Silica, Katy, TX), VCAS-160 (Vitro Minerals, Jackson, TN, USA), and Ball Clay (The Ceramic Shop, Norristown, PA, USA). These materials were chosen as references because they were expected to contain fundamentally different types of silica particles that might be representative of real silica particles in RCMD. Specifically, the Min-U-Sil 5 and Sil-Co-Sil are high-purity quartz products (i.e., to represent pure silica particles); the VCAS-160 is a high-purity aluminosilicate glass product (i.e., to represent silica particles with homogeneous aluminum impurity); and the Ball Clay is an aluminosilicate (kaolin- and mica-rich) product with moderate quartz content (i.e., such that the silica particles might represent silica with surface-associated aluminosilicates). (To explain the expected association between silica and aluminosilicates in RCMD, it must be noted that silicates, predominantly aluminosilicates, are typically the most common minerals in RCMD; and, like silica, silicates are primarily sourced from the rock strata being mine along with the coal or drilled for roof bolting [4]. Thus, analysis of silica in RCMD samples typically must account for the presence of aluminosilicates.)

Respirable samples of each of the four reference materials were generated in the same enclosure and with the same sampling equipment as used for the samples from mine dust-source materials. The respirable silica image library includes particles from all four reference materials.

### 4.2 SEM-EDX work

Scanning electron microscopy with energy dispersive X-ray spectroscopy (SEM-EDX) was used to identify, image and analyze silica particles in all 61 samples shown in Table 1. An effort was made to find at least four silica particles in each sample, however this was not always possible given the relatively low silica content in some samples (e.g., those generated from rock dust products). Total time for SEM-EDX work was generally limited to 1 h per sample.

The SEM-EDX system included a FEI Quanta 600 FEG environmental scanning electron microscope (ESEM) (Hillsboro, OR, USA) equipped with a backscatter electron detector (BSD) and a Bruker Quantax 400 EDX spectroscope (Ewing, NJ, USA), and Bruker Esprit software (version 1.9.4).

#### 4.2.1 Sample preparation

All SEM-EDX work was performed directly on the PC filter samples. For the prior study [1,2], the samples had already been prepared as follows: From each 37-mm filter, a 9-mm subsection was cut using a clean, stainless-steel trephine. The subsection was mounted to an SEM stub, and sputter coated with a thin layer of Au/Pd to render it electrically conductive.

#### 4.2.2 Silica particle identification

SEM-EDX work was performed in two steps on each sample. First, the sample was scanned to identify silica particles. For this, the SEM was operated under high vacuum, with an accelerating voltage of 15 kV, 12.5 mm working distance, 5.5 spot size, 92.5% brightness, and 60–70% contrast. In each field of view at 5000× magnification, elemental maps (Si and Al) were generated as a screening tool to for silica particles. For any particles that appeared to be Si-dominant, point-analysis was used to collect the elemental spectrum and determine normalized atomic % for eight elements (C, O, Al, Si, Ca, Mg, Fe and Ti). Consistent with the prior study of silica characteristics [2], particles were identified as silica if they had: a) Si/(Si+Al)>70% and b) sufficiently low content of Ca, Mg, Fe and Ti, since these since these elements would indicate silicates other than aluminosilicates.

#### 4.2.3 Silica particle imaging and analysis

The second step for the SEM-EDX work was to image and analyze each of the silica particles identified during the first step. For this, the magnification was increased to 10,000–20,000×, depending on the size of the particle. Then, micrographs with elemental maps (Si and Al only) and spectra were captured at both 5 and 20 kV. These data were tabulated (along with sample identifiers) to compile the *Respirable silica particle image library* table (per the format illustrated in Table 2). Additionally, a table was compiled of *Normalized atomic % values* for each particle at 5 and 20 kV for all eight elements included in the analysis.

It should be explained that data collection at both low and high accelerating voltage was done to enable interpretation of the particle surface versus the core, since the 5 kV electron beam is relatively shallow whereas the 20 kV beam is relatively deep. This approach was previously developed and used to explore respirable silica particles [5,6], and it was specifically used in the prior study of respirable silica characteristics in RCMD [2] which prompted the current effort to build an image library. Table 3 shows example images from the library for silica particles identified in the samples generated from reference materials. As expected, the silica particles from Min-U-Sil 5 and Sil-Co-Sil products appear to have negligible Al content at 5 and 20 kV; the silica particles from the VCAS-160 product have a minor but consistent fraction of Al at both 5 and 20 kV; and the silica particles from the Ball Clay product appear to have more Al at 5 kV (i.e., on the particle surface) than at 20 kV (i.e., within the core).

**Table 3:** Example SEM images (captured at 5 and 20 kV) with overlaid Si (green) and Al (red) maps showing silica particles in respirable dust samples generated from reference materials: a) and b) Min-U-Sil 5, c) and d) Sil-Co-Sil, e) and f) VCAS-160, g) and h) Ball Clay. Normalized atomic % values for Si and Al are also shown; in images with multiple silica particles, the values are reported for the particle in the center of the image. (Images with reddish background indicate that Al signal across the entire mapped field was essentially noise, i.e., Al content in the particle(s) was no higher than in the background.)

## Ethics Statements

The authors confirm this dataset has been collected, curated, and reported in compliance with ethical publishing requirements of the journal and the publisher. This work did not involve human or animal subjects.

## CRediT authorship contribution statement

**Çiğdem Keleş:** Conceptualization, Methodology, Investigation, Data curation, Validation, Writing – original draft, Writing – review & editing. **Emily Sarver:** Conceptualization, Writing – original draft, Writing – review & editing, Resources, Project administration, Funding acquisition.

## Data Availability

Respirable silica particles in coal mine dust: image library (Original data) (Mendeley Data)

## Footnotes

1 Prior research has indicated that most silica particles in RCMD are quartz; for example [1] shows good correlation between estimates of silica mass % derived from SEM-EDX data and measurements of alpha-quartz by FTIR. However, it should be noted that SEM-EDX cannot be used to determine crystallinity of particles. Therefore, this paper refers to silica particles, rather than crystalline silica or quartz particles.

## References

[1] Keles C., Pokhrel N., Sarver E. (2022). A study of respirable silica in underground coal mines: sources. Minerals.

[2] Keles C., Sarver E. (2022). A study of respirable silica in underground coal mines: particle characteristics. Minerals.

[3] International Organization for Standardization (1995).

[4] Sarver E., Keles C., Afrouz S.G. (2021). Particle size and mineralogy distributions in respirable dust samples from 25 US underground coal mines. Int. J. Coal Geol..

[5] Wallace W.E., Harrison J.C., Grayson R.L., Keane M.J., Bolsaitis P., Kennedy R.D., Wearden A.Q., Attfield M.D. (1994). Aluminosilicate surface contamination of respirable quartz particles from coal mine dusts and from clay works dusts. Ann. Occup. Hyg..

[6] Harrison J.C., Brower P.S., Attfield M.D., Doak C.B., Keane M.J., Grayson R.L., Wallace W.E. (1997). Surface composition of respirable silica particles in a set of US anthracite and bituminous coal mine dusts. J. Aerosol. Sci..

